# Premature Termination Codon of *1Dy12* Gene Improves Cookie Quality in Ningmai9 Wheat

**DOI:** 10.3389/fpls.2022.835164

**Published:** 2022-05-12

**Authors:** Guangxiao Liu, Yujiao Gao, Huadun Wang, Yonggang Wang, Jianmin Chen, Pingping Zhang, Hongxiang Ma

**Affiliations:** ^1^Jiangsu Co-innovation Center for Modern Production Technology of Grain Crops/Jiangsu Key Lab of Crop Genomics and Molecular Breeding, Yangzhou University, Yangzhou, China; ^2^Co-innovation Center for Modern Crop Production Co-sponsored by Province and Ministry, Jiangsu Academy of Agricultural Sciences, Nanjing, China

**Keywords:** wheat, quality, mutant, HMW-GS, cookie

## Abstract

The area between middle and lower reaches of the Yangtze River is the largest region for soft wheat production in China. In soft wheat breeding, the lack of germplasm with desirable quality for end-use products is a barrier. Ningmai9 is the main variety of soft wheat planted in this area. To create germplasm with better quality and yield potential than Ningmai9, mutants of HMW-GSs in Ningmai9 induced by ethylmethanesulfonate (EMS) were obtained. SDS-PAGE showed that two mutants, md10 and md11, were HMW-GS 1Dy deletions. DNA sequencing confirmed that one mutation was caused by a C/T substitution, resulting in the change of CAA encoding glutamine into the termination codon TAA, and another mutation was due to a G/A substitution in the central repetitive domain of the coding region, causing TGG encoding tryptophan to become the termination codon TGA. The premature termination codon of the *1Dy12* gene affected the expression of *1Dy12* and kept the mRNA at a lower transcription level during the kernel development stage in comparison with the wild type. HMW-GS 1Dy12 deletion mutants decreased the content of HMW-GSs and glutenin macropolymers, mixograph envelope peak time and TIMEX width, water solvent retention capacity (WSRC), and lactic acid solvent retention capacity (LASRC). In the HMW-GS 1Dy12 deletion lines, the sugar-snap cookie diameter was 8.70–8.74 cm, which was significantly larger than that in the wild type of 8.0 cm. There were no significant differences in spike number, kernel number, thousand kernel weight, and yield between the deletion lines and wild type. Overall, the study indicated that the knockout of the *HMW-GS* gene induced by EMS is an effective way to improve wheat quality, and deletion mutants of HMW-GS 1Dy12 decrease gluten strength and increase sugar snap cookie diameter without yield penalty in Ningmai9 wheat.

## Introduction

Wheat is a primary staple food crop worldwide and has specific quality requirements for particular end uses, such as bread, cake, biscuit, and noodle. Improving processing quality is one of the major targets in wheat breeding. Hard and soft wheat are two basic market classes of wheat. Hard wheat flour usually has high protein content, strong gluten strength, and high water absorption, which enhances yeast growth and increases bread volume, whereas soft wheat flour has low protein content, less water absorption, and a small particle size distribution, which improves dough flow and product texture associated with cookie, cake, and cracker (Souza et al., 2002).

Wheat grain protein is divided into gluten and non-gluten fractions, and the wheat processing quality mainly depends on the gluten fractions (Ma W. et al., 2019). Wheat gluten is regarded as the major determinant of dough elasticity and viscosity, largely affecting the processing properties of wheat flour suitable for leavened bread and other bakery products (Shewry, 2019). Wheat gluten is comprised of many types of proteins, which are traditionally categorized into two groups, namely, glutenins and gliadins, in approximately equal amounts. Glutenins are assembled into polymers stabilized by interchain disulfide bonds, whereas gliadins are present as single polypeptide chains (Shewry and Halford, 2002; Lambourne et al., 2010). The polymeric glutenins include high molecular weight glutenin subunits (HMW-GSs) and low molecular weight glutenin subunits (LMW-GSs) (Rasheed et al., 2014). HMW-GSs are at relatively low levels in wheat grains, accounting for 5–10% of the storage proteins (Branlard and Dardevet, 1985; He et al., 2005); however, they provide disulfide-bonded backbones in the gluten network and are recognized as the most important components in determining the strength and elasticity of wheat gluten (Shewry et al., 2003; Li et al., 2021). Three homoeologous alleles *Glu-A1*, *Glu-B1*, and *Glu-D1* encode HMW-GSs at the *Glu-1* loci on the long arms of group 1 chromosomes in wheat (Payne et al., 1981). There are two tightly linked genes at each HMW-GS locus, encoding an x-type (80–88 kDa) and a y-type (67–73 kDa) subunits. Theoretically, six different HMW-GSs are involved in wheat, but in fact, there are only three to five HMW-GSs in most wheat cultivars (Payne, 1987). The genes of *1Bx*, *1Dx*, and *1Dy* are present in most cases, whereas the genes of *1Ay* are usually silenced (Roy et al., 2018; Yu et al., 2019). Each *HMW-GS* gene has multiple alleles, which constructs different composition among different varieties of wheat and other species of *Triticeae* (Liu et al., 2005). So far, more than 30 alleles of *HMW-GS* genes have been isolated, and the sequences of HMW-GSs are highly conserved and share the same primary structure composed of a signal peptide, an N-terminal domain, a central repetitive domain, and a C-terminal domain. The majority of x-type subunits possess four conserved cysteine residues, and the y-type subunits generally contain seven conserved cysteine residues. Different subunits possess different numbers of cysteines (Rasheed et al., 2014; Ma W. et al., 2019). The cysteine residues in both x-type and y-type subunits form glutenin macropolymers (GMPs) in the gluten complex in dough by intermolecular disulfide bonding. Based on SDS extractability, the GMP complexes are divided into SDS-unextractable polymeric protein (UPP) and SDS-extractable polymeric protein (EPP) (Ma W. et al., 2019). UPP contains larger GMP complexes, while EPP is composed of smaller GMP complexes. The ratio of UPP% is positively correlated with gluten and dough strength (Shewry and Tatham, 1997).

High molecular weight glutenin subunits and their combinations affect the physical and physicochemical properties of dough. Some subunit combinations, such as 1Dx5 + 1Dy10, 1Bx7 + 1By8, and 1Bx17 + 1By18, are desirable subunits for bread-making quality by contributing to superior dough strength and elasticity, whereas other subunit combinations, such as 1Dx2 + 1Dy12, 1Bx20, and 1Bx7 + 1By9, decrease dough strength (Shewry et al., 1992; Yan et al., 2009; Liu et al., 2016; Guo et al., 2019; Jiang et al., 2019).

The main biological function of storage protein is to provide nutrition and energy sources for seed germination and seedling growth in wheat. Mutations of *HMW-GS* gene silencing are not fatal for the plant, so the selection pressure on these genes has less effect on the growth and development of wheat than that on functional genes (Liu et al., 2008). As a result, these genes can be modified to create more mutations to improve the processing quality of wheat breeding. However, as shown in previous reports, the effect of silencing HWM-GSs on end-use products, especially in soft wheat, was not consistent. The deletion of all HMW-GSs significantly reduced the dough strength, GMP content, and bread-backing quality (Zhang Y. et al., 2018). The loaf volumes of the five knockout mutants with the deletion of one or two HMW-GS genes in *1Ax1*, *1Bx14*, *1By15*, *1Dx2*, and *1Dy12* were significantly smaller than those of the wild-type Xiaoyan54 (Li et al., 2015). Wang et al. (2017) found that the *Glu-D1* locus had a greater effect on bread-processing quality than the loci of Glu-A1 and Glu-B1, and 1Dx2 had a stronger function than 1Dy12 in promoting functional glutenin macropolymers by comparing two series of genetic mutants. The contribution of each of the HMW-GS to the bread-processing quality of deletion lines missing the loci *Glu-A1*, *Glu-B1*, and *Glu-D1* created by the ion beam methods showed that the genetic effects of the *Glu-1* locus on gluten functionality were *Glu-D1* > *Glu-B1* > *Glu-A1* (Yang et al., 2014). Ram et al. (2007) reported that double nulls of x-type and y-type subunits at the *Glu-D1* locus were associated with reduced gluten strength and suitable for biscuit making in the Indian wheat landrace Nap Hal, which might be useful in soft wheat breeding. Two lines containing Glu-Bx17 and By18 HMW-GSs and lacking *Glu-A1* and *Glu-D1* significantly increased tortilla diameters (Mondal et al., 2008). The deletion lines decreased UPP, HMW/LMW, and dough extensibility compared with non-deletion lines (Zhang P. et al., 2014). Gao et al. (2018) found that the dough development and stability time were significantly reduced in near-isogenic lines with the deletion of 1Ax1 or 1Dx2, but the uniformity of the microstructure in wheat was increased. Knocking-out 1Bx7 or 1By9 significantly decreased the accumulation of gluten proteins and weakened dough strength, which led to an inferior sponge cake quality (Chen et al., 2019). Recent research showed that 1Dy missing had a negative impact on the quality of bread, sponge cake, and biscuit (Chen et al., 2021).

China is one of the world’s largest wheat producers, with output reaching more than 130 million tons in 2020, according to the statistics of Ministry of Agriculture and Rural Affairs of the People’s Republic of China. To meet the market demand, Chinese scientists began focusing on wheat quality improvement in the last 30 years (Wang et al., 2018). Many hard white wheat cultivars with good end-use quality were released in northern China (Zhang et al., 2007; Li et al., 2019). However, the improvement in soft wheat quality was less than that in hard wheat (Yao et al., 2014). Soft wheat consumption has significantly increased, reaching more than six million metric tons annually in China. Soft wheat quality improvement is highlighted of great importance in production, especially in the middle to lower reaches of the Yangtze River for winter wheat growing. This is the best region for soft red winter wheat production in China (Zhang et al., 2012).

A lack of germplasm with desirable quality for end-use products is a barrier to soft wheat breeding in China. Most soft wheat varieties with good quality from the United States or Australia are hard to use in direct wheat breeding and production in China as the yield potential and the resistance to biotic or abiotic stress are not adapted to the local ecological regions in China. Zhang et al. (2007) evaluated the quality of soft wheat varieties in China and found that only three varieties were considered good soft wheat with acceptable cookie-making qualities. Ningmai9 is the major variety used in the middle and lower reaches of the Yangtze River, which has desirable agronomic traits and has had stable soft wheat quality across variable regions for many years. In addition, Ningmai9 is a founder parent in wheat breeding and has derived 23 released varieties for wheat production so far (Ma H. et al., 2019).

Soft wheat flour is suitable for producing cookie, cake, and cracker. It usually needs smaller flour particles, higher amylose content, and fewer gluten proteins than hard wheat. The variation of gluten protein in soft wheat weakens the gluten network in dough. There were significant positive correlations between high molecular weight polymeric protein fractions and single kernel hardness index as well as mixograph water absorption/tolerance, but a negative correlation with break flour yield, cookie diameter, and cake volume in soft wheat (Ohm et al., 2009). To meet the urgent demand for soft wheat breeding in China, we developed a set of deletion mutants of HMW-GSs in Ningmai9 (Zhang J. et al., 2014). This research is expected to identify the molecular mechanisms and functions of HMW-GS deletion mutants and their effects on cookie quality, so as to evaluate their yield potential and provide useful materials in the breeding and production of soft wheat.

## Materials and Methods

### Plant Materials and Field Experiment Design

Wheat seeds were treated by 0.4% EMS and sowed in the experiment station of Jiangsu Academy of Agricultural Sciences (Zhang J. et al., 2014). M_2_ and M_3_ seeds of EMS-mutagenized population were harvested and screened for the variation of HMW-GS. The wild type is a soft wheat variety, Ningmai9, which was released by the Jiangsu Academy of Agricultural Sciences using an intervarietal cross of Yangmai 6/Xifeng. The HMW-GS composition of Ningmai9 consists of 1Ax, 1Bx7, 1By8, 1Dx2, and 1Dy12. After three generations of self-pollination and selection, we planted M_7_ seeds of mutant lines and the wild type at the experimental station. The field experiment was designed as a randomized complete block experiment for one factor. Each treatment had a plot size of 5 m^2^, with a seed density of 2.4 × 10^6^ ha^–1^ and a row spacing of 25 cm. There were three replications in each treatment. The spike number was measured at the maturity stage. The yield in each plot was calculated after harvest. Other agronomic traits, including plant height, kernel number per spike, and thousand kernel weight, were examined using an average of 10 plants randomly collected from each plot. The seeds were harvested and mixed up for a grain quality test in each plot.

Five immature kernels were collected from mutants and wild types in the middle part of the selected spikes at 7, 11, 14, and 21 days after anthesis. The collected samples were immediately frozen in liquid nitrogen and then used to extract RNA for gene expression analysis.

### SDS-Polyacrylamide Gel Electrophoresis Assay for Seed Storage Proteins

High molecular weight glutenin subunits were separated by SDS-PAGE according to the method described previously (Khan et al., 1989). In brief, a 40 mg grain was ground and defatted with chloroform in a 2 ml Eppendorf tube. The tube with sample was subsequently mixed with 1 ml of extraction buffer, which contained 62.5 mmol l^–1^ Tris–HCl (pH6.8), 10% glycerol, 2% SDS, and 5% β-mercaptoethanol. The mixture was incubated at room temperature for 30 min with unremitting shaking, and then incubated at 90°C for 5 min. Thereafter, the sample was centrifuged at 8,000 × *g* for 15 min. The supernatant was used for SDS-PAGE.

The acrylamide concentration was 10 and 4% in the resolving gel and stacking gel, respectively. In total, 25 ml of glutenin extract was loaded in each lane for 10 h of electrophoresis. The resolving gel was then stained with 0.05% Coomassie Brilliant Blue R250 for 24 h, followed by destaining in distilled water for 48 h until the bands were clearly distinguished. The destained gel was then scanned using VersaDoc Model 5000 Imaging Systems and analyzed for the preliminary content of each HMW-GS with Quantity 1-D Analysis Software (Bio-Rad Laboratories, Irvine, CA, United States). Afterward, each band was cut separately from the gel and placed in an Eppendorf tube. A 500–1,000 ml mixture of 50% isopropyl alcohol containing 3% SDS was added to the tube according to the content of each band, and then the tube was placed for incubation at 37°C for 24 h until the gel cleared. The extraction was then detected at a wavelength of 595 nm using a UV-2401 Shimadzu spectrophotometer.

The standard protein (116 kD, Sigma) was dissolved in 20, 30, and 40 ml volumes and loaded in three lanes on the same gel, and the standard curve was obtained according to the content of the standard protein extracted and measured following the above steps for quantification. Each HMW-GS of collected samples was then quantified based on the standard curve.

### DNA Extraction and PCR Amplification

Genomic DNA was extracted from the leaves of a 3-week-old seedling using the cetyl trimethyl ammonium bromide (CTAB) method. The forward primer (5′-GGCTAACAGACACCCAAAC-3′) and reverse primer (5′-TGTGAACACGCATCACGT-3′) for specific detection of 1Dy12 were designed according to published DNA sequences to amplify the complete coding sequence in mutants and wild type. TksGflex DNA polymerase (TaKaRa, Shanghai, China) was used for the amplification. In each reaction, 500 ng of genomic DNA, 1× Gflex buffer, 1 μl of Tkspolymerase, and a concentration of 0.2 μM of each primer were added to the tube in a volume of 50 μl of double-distilled water. The PCR was run in a Bio-rad T100 thermal cycler starting with 2 min at 94°C, followed by 35 cycles of denaturing at 98°C for 10 s, annealing for 15 s at 60°C, and extension at 68°C for 90 s, with a final extension of 5 min at 68°C. The PCR products were separated on 1.0% agarose gels at 170 V for 10 min.

### *1Dy* Gene Cloning and Sequencing

The target DNA fragments were cut from the agarose gel and purified using the TIAN gel Midi Purification Kit (TIANGEN BIOTECH, Beijing, China). Purified regenerants were then ligated into the pEASY-Blunt Simple Cloning Kit (TransGen Biotech, Beijing, China) and transformed into *Escherichia coli* Trans1-T1 Phage Resistant Chemically Competent Cells (TransGen Biotech, Beijing, China). The transformed cells were tiled on ampicillin LB medium containing IPTG and X-Gal for culturing at 37°C for 12–16 h. The positive clones were amplified to test for the target fragment, which was then sent to Takara Bio (Shanghai, China) for sequencing. DNAMAN version 6.0 software (Lynnon Biosoft, San Ramon, United States) and BioEdit version 7.1.3 software were used for assembling and aligning the gene sequences.

### RNA Extraction and Quantitative Reverse Transcription-PCR

Wheat kernels from all spikelets of spike were collected from mutants and wild types at different grain development stages from anthesis to harvest. The total RNAs of collected kernels were extracted using the Promega (Madison, WI, United States) SV total RNA isolation system (Promega, United States). The RNA PCR Kit (AMV) version 3.0 (Takara Bio, Shanghai, China) was used for synthesizing the first-strand cDNAs. In addition, 10 μl 2× RealStar Green Fast Mixture with ROXII, 0.6 μl primer mix (10 μM) each for 1Dy12, 1 μl first-strand cDNA, and 8.2 μl ddH_2_O were added to a 20 μl reaction volume for qRT-PCR. The reactants were amplified using an ABI PRISM 7500 Real-Time PCR System (ABI, Redwood City, CA, United States) starting with 2 min at 94°C, followed by 50 cycles of denaturing at 95°C for 20 s, annealing for 20 s at 55°C, and extension at 72°C for 20 s. The tubulin gene was used as an endogenous control gene. Data from individual runs were collated using the 2^–ΔΔCT^ method (Livak and Schmittgen, 2001).

### Polymeric Protein Examined by High-Performance Liquid Chromatography

The SDS extractable and unextractable proteins were extracted, and HPLC analysis was conducted according to the method described previously (Larroque et al., 2000). A total of 10 mg of flour sample in a 1.5-ml microfuge tube was mixed with 1 ml of 0.5% SDS in 0.05 M of phosphate extraction buffer (PEB). The mixture was subjected to a 1 min vortex and centrifuged at 14,000 rpm for 15 min. After centrifugation, the SDS-soluble fraction was separated on supernatant and subsequently decanted into a clean 1.5-ml microfuge tube. The remaining pellet in the microfuge tube was mixed with 1 ml of PEB for resuspending and subjected to a 30 s sonication. The sample was then subjected to centrifugation for 15 min at 14,000 rpm. The supernatant was an SDS-insoluble fraction. The SDS-insoluble fraction was transferred to a clean 1.5-ml microfuge tube. Both SDS-soluble and insoluble fractions were subjected to an 80°C water bath for 2 min to inactivate proteases and then filtered into 2 ml glass vials for HPLC. The percentage of SDS-unextractable polymeric protein in total polymeric protein (UPP/UPP + EPP) was calculated based on the ratio of the peak area for unextractable polymeric protein relative to the total area of both the extractable and unextractable polymeric protein.

### Quality Testing of Grain, Flour, and Sugar Snap Cookie

Grain protein content (GPC) was determined using a near-infrared reflectance spectroscopy analyzer (NIR) (Perten DA 7200, Perten Instruments, Huddinge, Sweden) following AACC method 39-10 (AACC 2000). Wet gluten content was examined based on the National Standards of China (GB/T5506.2-2008 and GB/T5506.4-2008). The results were expressed on a 14% moisture basis.

Hardness and diameter of the grain were tested using the Single Kernel Characterization System (SKCS 4100, Perten Instruments Co., Ltd., Stockholm, Sweden) of AACC method 55-31.03 (AACC 2000).

For milling, grain samples were cleaned and adjusted to 14.5% moisture for 18 h. Grain samples were then roller-milled to straight-grade flours according to AACC 26-31 using a Buhler Experimental Mill [MLU-202, Buhler Equipment Engineering (Wuxi) Co., Ltd., Jiangsu, China]. The yield of straight-grade flour was about 70%.

Solvent retention capacity (SRC) of flour, including water solvent retention capacity (WSRC), sodium carbonate solvent retention capacity (SCSRC), lactic acid solvent retention capacity (LASRC), and sucrose solvent retention capacity (SUSRC), were measured according to AACC method 56-11 (AACC 2000). Dough rheological properties were determined using mixograph according to AACC 54-40.02. Sugar snap cookie was processed according to AACC 10-52, and the cookie diameter was noted.

### Statistical Analysis

A significant difference in the tested characters between deletion mutants and wild type was determined using analysis of variance (ANOVA) and Student’s *t*-test. The criterion for statistical significance was set at *P* < 0.01 and *P* < 0.05.

## Results

### Identification of 1Dy12 Mutants

**FIGURE 1:**
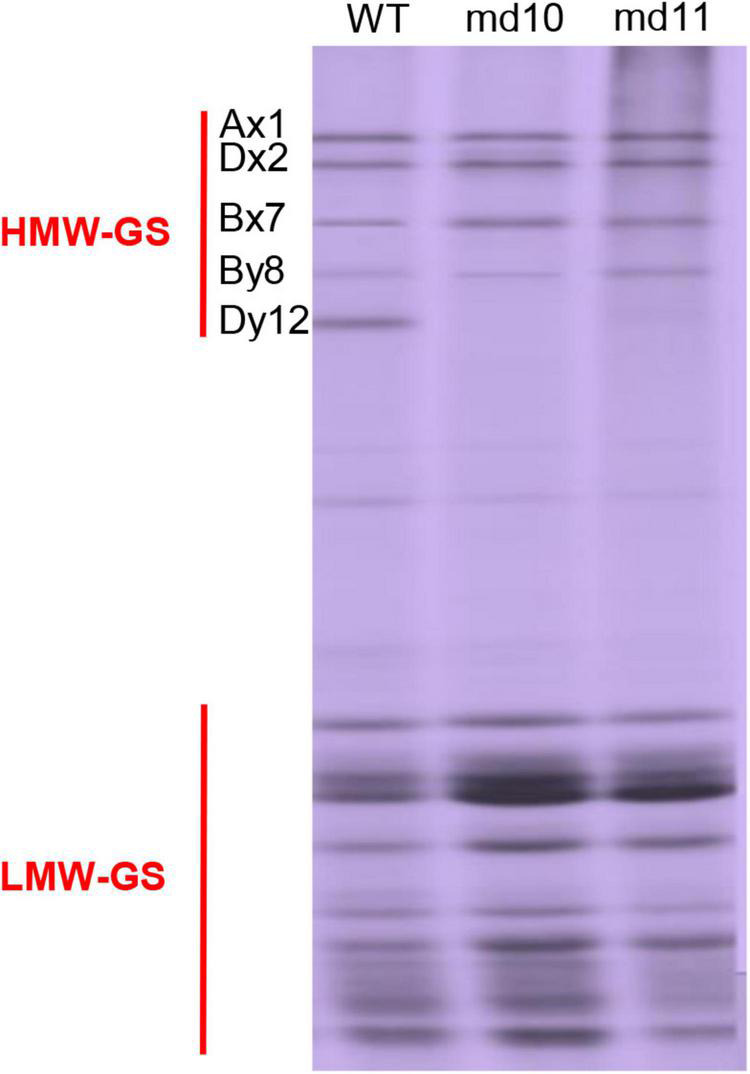
SDS-PAGE pattern of HMW-GSs and LMW-GSs in the wild type and mutants, md10 and md11.

SDS-PAGE analysis showed that the composition of HMW-GSs in the soft wheat Ningmai9 was 1Ax1, 1Bx7, 1By8, 1Dx2, and 1Dy12. In total, 3,781 lines mutagenized from the Ningmai9 by EMS were used to screen for mutants lacking the HMW-GSs. Two independent mutants (md10 and md11) with 1Dy12 deletion were identified, but the patterns of LMW-GSs and other HMW-GSs were unchanged when compared with the wild type (Figure 1).

### Sequencing of *1Dy12* Gene in Wild Type and Mutants

**FIGURE 2:**
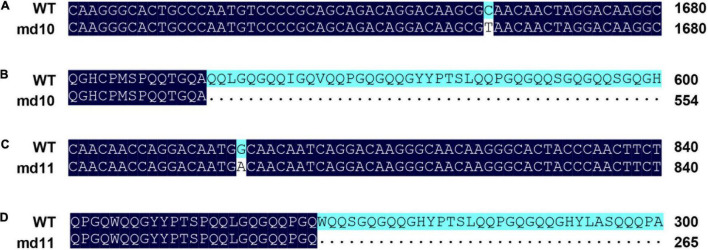
Alignment analysis of the *1Dy12* sequences in mutant md10, md11, and wild type. **(A)** Partial DNA sequence alignment of *1Dy12* of md10 and wild type, **(B)** partial protein sequence alignment of 1Dy12 of md10 and wild type, **(C)** partial DNA sequence alignment of *1Dy12* of md11 and wild type, and **(D)** partial protein sequence alignment of 1Dy12 of md11 and wild type.

A pair of specific primers designed outside the coding region of the *1Dy12* gene was amplified, and a target band of approximately 2.5 kbp was amplified in mutants and wild type (Supplementary Figure 1). The sequences of coding regions of *1Dy12* in mutants and wild type were analyzed. The open reading frames of *1Dy12* in wild type were 1,980 bp in length with the initiation codon and two duplicate termination codons, encoding 658 amino acid residues (Supplementary File 1). The sequence comparison between the mutants and wild types indicated that each mutant has single base substitutions, respectively. The mutation site of md10 occurred at 1,663 bp with a substitution of C/T, which changed the codon CAA of glutamine to the termination codon TAA, substitution located on the fifth amino acid residue of the central repetitive domain (TGQAQQ) (Figures 2A,B). A G/A substitution occurred at 798 bp in mutant md11, causing the tryptophan codon TGG to become the termination codon TGA; this substitution occurred at the W position of the PGQWQQ in the central repetitive domain (Figures 2C,D).

### Expression of *1Dy12* Gene in Grain Development Stages

**FIGURE 3:**
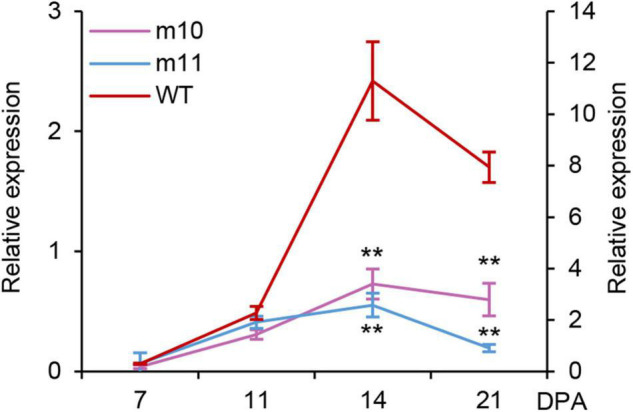
Expression profiling of the *1Dy12* gene at a different developmental stage in mutants md10, md11 (y-axis on the left), and wild type (y-axis on the right) by qRT-PCR. The double asterisks represent significant differences determined by Student’s *t*-test at *P* < 0.01.

Total RNA was extracted and reverse transcribed to cDNA from the grains of mutants and wild type at different days after anthesis (DPA) and used for gene expression analysis to clarify if the *1Dy12* gene at the *Glu-D1* locus was normally expressed at transcriptional and translational levels in mutants during the course of grain development. The expression profiles of the *1Dy12* gene in mutants and Ningmai9 were determined using qRT-PCR. The gene expression of *1Dy12* at least started at 7 DPA, reached its highest level with the relative expression of 11.3 at 14 DPA, and then declined as grain-filling progressed in the wild type of Ningmai9. The expression of *1Dy12* decreased across all developmental stages in both mutants of md10 and md11 in comparison with wild type; the values of relative expression ranged from 0.03 to 0.72 and from 0.07 to 0.55 in md10 and md11, respectively, which implied that the expression of *1Dy12* in mutants was significantly suppressed and resulted from a premature termination codon (Figure 3).

### Grain Characters and Storage Protein in Wild Type and Mutants

**FIGURE 4:**
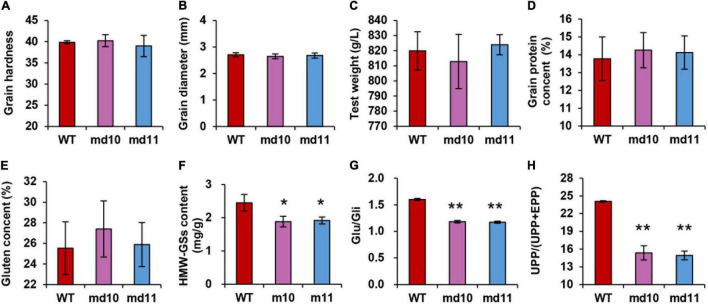
Values of **(A)** grain hardness, **(B)** grain diameter (mm), **(C)** test weight, **(D)** grain protein content (%), **(E)** grain gluten content (%), **(F)** HMW-GS content (mg g^–1^), and **(G)** Glu/Gli and **(H)** UPP/(UPP + EPP) in wild type and mutants (md10 and md11). Data represent mean ± standard deviation. Single and double asterisks show significant differences at *P* < 0.05 and *P* < 0.01 using Student’s *t*-test, respectively.

Grain hardness and diameter were detected by SKCS. There was minimal difference between wild type and 1Dy12 deletion mutants in both characters. The grain hardness and diameter of 1Dy12 deletion mutants were ranged from 38.97 to 40.23 and from 2.65 to 2.68 mm, respectively, while those of the wild type were ranged between 39.85 and 2.71 mm (Figures 4A,B). Test weight in mutants was not significantly different from that in wild type, too (Figure 4C). Similarly, no significant differences were detected in grain protein content and wet gluten content. The grain protein content and wet gluten in 1Dy12 deletion mutants ranged from 14.13 to 14.26% and from 25.88 to 27.40%, respectively, while those of the wild type were 13.77 and 25.53% (Figures 4D,E). In comparison with the wild type, the total amount of HMW-GSs in mutant md10 and md11 was decreased by 19.6 and 21.8% (Figure 4F). The comparison of storage protein components showed that Glu/Gli in md10 and md11 was 1.17–1.18. It was significantly lower than that in the wild type (1.6) (Figure 4G). UPP% in mutants and wild type were tested by high-performance liquid chromatography (HPLC). Compared with the wild type (24.07%), a significant decrease was observed in md10 (15.36%) and md11 (14.93%) (Figure 4H). The results indicated that the deletion of *1Dy12* has a negative impact on total HWM-GSs content, Glu/Gli, and UPP accumulation level, though other grain characters remain unchanged.

### Effect of the Lack of 1Dy12 on Solvent Retention Capacity

**FIGURE 5:**
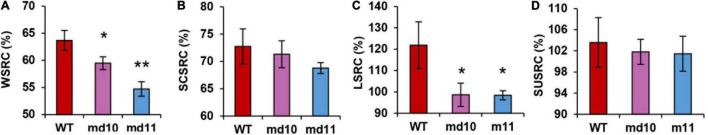
Solvent retention capacity, **(A)** WSRC, **(B)** SCSRC, **(C)** LASRC (%), and **(D)** SUSRC (%) of flour in wild type and mutants (md10 and md11). Data represent mean ± standard deviation. Single and double asterisks show significant differences at *P* < 0.05 and *P* < 0.01 using Student’s *t*-test, respectively.

The SRC test can describe the intrinsic characteristics of flour components and is widely used in soft wheat breeding. The comparison of SRC value between *1Dy12* deletion mutants and wild type showed that the effect varied among different SRC components. The WSRC in md10 and md11 was 59.48 and 54.72%, respectively, a significant decrease in comparison with that in wild type (63.68%) (Figure 5A). LASRC in *1Dy12* deletion mutants was ranged from 98.41 to 98.65%, and significantly lower than that in wild type (121.85%) (Figure 5C). There was a small difference between wild type and *1Dy12* deletion mutants in SCSRC and SUSRC, and the variation ranged from 68.79 to 72.76% and from 101.46 to 103.58%, respectively (Figures 5B,D).

### Effect of the Lack of 1Dy12 on Processing Quality

**FIGURE 6:**
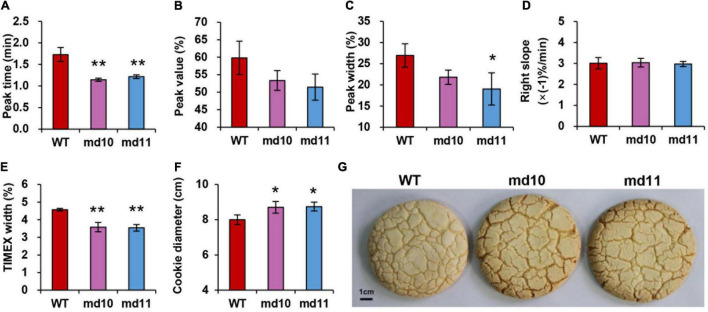
Dough rheological properties, **(A)** peak time (min), **(B)** peak value (%), **(C)** peak width (%), **(D)** right slope (% min^–1^), **(E)** TIMEX (%) using mixograph, **(F)** cookie diameter in wild type and mutants (md10 and md11), and **(G)** top view of sugar snap cookie of wild type (middle) and mutants, md10 (left) and md11 (right). Data represent mean ± standard deviation. Single and double asterisks show significant differences at *P* < 0.05 and *P* < 0.01 using Student’s *t*-test, respectively.

Processing quality parameters related to dough rheological properties were characterized, and comparisons between 1Dy12 deletion mutants and wild type were made. Dough rheological properties were detected using mixograph. There were significant differences in peak time and TIMEX width between 1Dy12 deletion mutants and wild type. The peak time and TIMEX width were 1.73 min and 4.57% in wild type and decreased to 1.15–1.22 min and 3.54–3.58% in deletion mutants, respectively (Figures 6A,E). No significant differences in peak value, peak width, and right slope were found between deletion mutants and wild type (Figures 6B–D). Sugar snap cookies with flours of *1Dy12* deletion mutants and wild type were made, and their diameter is shown in Figure 6F. The wild type had a cookie diameter of 8.00 cm, and the deletion mutants had an increased spreading ability and more width compared with those of the wild type, which had a cookie diameter of 8.70–8.74 cm. The result suggested that 1Dy12 deletion in Ningmai9 demonstrated the superior quality of sugar snap cookies (Figure 6G).

### Yield Related Traits in Wild Type and Mutants

**FIGURE 7:**
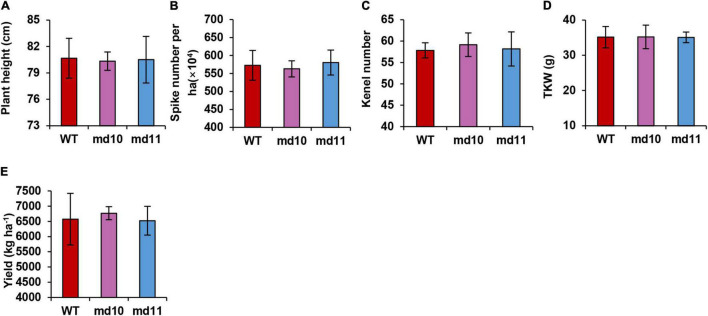
Yield related characters, **(A)** plant height (cm), **(B)** spike number per ha, **(C)** kernel number per spike, **(D)** TKW (g), and **(E)** yield (kg ha^–1^).

The 1Dy12 deletion mutants, md10 and md11, and wild type were sown in the field at the end of October 2017 and harvested in early June in 2018. Morphological traits were studied in mutants and wild type. The 1Dy12 deletion mutants md10 and md11 had similar performance to wild type. The plant height, spike number per ha, kernel number per spike, thousand kernel weight, and yield per ha of 1Dy12 deletion mutant were 80.33–80.50 cm, 563.0–580.3 × 10^4^, 57.83–59.17, 35.08–35.22 g, and 6,520–6,770 kg ha^–1^, respectively, while those of wile type were 80.67 cm, 572.7 × 10^4^, 57.83, 35.16 g, and 6,573 kg ha^–1^ (Figures 7A–E). The results indicated that 1Dy12 deletion mutants possessed the same yield potential as wild type.

## Discussion

The quality of end-use products is affected by different subunits of wheat HMW-GSs both individually and cooperatively (Delcour et al., 2012). Generally, the absence of an individual or combination of HMW-GSs decreases the gluten strength and elasticity and has negative effects on bread-processing quality (Li et al., 2015; Liu et al., 2016; Wang et al., 2017; Gao et al., 2018; Zhang Y. et al., 2018). However, for the end-use of soft wheat, the effect of the deletion of HMW-GSs was inconsistent with the previous studies. Tuncil et al. (2016) reported that the presence of 1Bx7 and 1By9 at Glu-B1 and the absence of Glu-A1 and Glu-D1 consistently enlarged the diameter and kept favorable flexibility during storage for tortillas. Significantly higher cookie diameter and lower cookie height were found in near-isogenic lines of double null in Glu-A1 and Glu-D1 and single null in Glu-D1 compared with their recurrent parent Yangmai18 (Zhang X. et al., 2018). However, Chen et al. (2019) found that 1Bx7 and 1By9 make important contributions to gluten functionality, and the deletion of 1Bx7 or 1By9 in mutants resulted in weaker dough strength and inferior sponge cake performance. It was also reported that a wheat somatic variation of the 1Dy12 deletion line showed an inferior sponge cake and biscuit in KN199 (Chen et al., 2021). The results show that the effects on the quality of end-use products are not only dependent on the function of different HMW-GSs but also related to the genetic backgrounds of wheat varieties. Clarifying the contributions of HMW-GSs in flour-processing quality is important in commercial varieties so that the effects of HMW-GSs can be efficiently utilized in wheat breeding. To improve soft wheat processing quality, we created deletion mutants of HMW-GSs from Ningmai9, a major soft wheat variety in the reaches of the middle to lower Yangtze River, using the EMS mutagenized method (Zhang J. et al., 2014). We found that the absence of 1Dy12 in both mutants, md10 and md11, had a positive effect on the quality of sugar snap cookies compared with the wild type, Ningmai9 (Figure 6). Yield-related traits in both mutants were similar to those in Ningmai9 in this study (Figure 7), which was similar to the report of Zhang X. et al. (2018), but not consistent with the result of the somatic mutant in KN199 (Chen et al., 2021). We speculated that different methods of mutant induction and the genetic background of the experimental materials might be responsible for such differences. Somatic variation may produce more variation loci than EMS; meanwhile, KL199 is hard winter wheat and Ningmai9 is soft spring wheat. However, md10 and md11 in this study could be useful for soft wheat production and breeding.

Deletion of HMW-GSs results from gene silencing. The gene silencing of HMW-GSs could be caused by the deletion of small chromosomal fragments and the alteration of promoter regions (Upelniek et al., 1995; Beshkova et al., 1998; Liu et al., 2016). Single base substitutions in the coding region sequences also cause gene silencing in the deletion of HMW-GSs in wheat, which usually results from premature termination codons (Chen et al., 2019, 2021). The gene silencing of *Glu-1Ay* in wheat is largely attributed to premature termination codons (Luo et al., 2018). There are two major mechanistic classes inducing gene silence, post-transcriptional gene silencing (PTGS), and transcriptional gene silencing (TGS) (Hammond et al., 2001). Chen et al. (2021) found that the gene silencing of *1Dy12* in the somatic mutant derived from KL199 was caused by PTGS, as RT-PCR and qRT-PCR results of *1Dy12* gene expression implied that this gene was normally expressed in the mutant through all grain developmental stages. In this study, DNA sequencing confirmed that one mutation caused by a C/T substitution in md10 would result in the change of CAA encoding glutamine into termination codon TAA, and the mutation of md11 is due to a G/A substitution within the coding region repeat sequence, making the tryptophan encoding TGG become the termination codon TGA (Figure 2). The gene expression of *1Dy12* in deletion mutants is different from that reported by Chen et al. (2021). In the research, the relative expression of *1Dy12* significantly decreased across all developmental stages in both mutants of md10 and md11 in comparison with the wild type (Figure 3). Therefore, we speculated that the gene silencing in md10 and md11 was caused by TGS. The result is similar to the research by Gao et al. (2021), in which a premature stop codon of *TaNAC019* affected not only its protein biosynthesis but also its mRNA production.

High molecular weight glutenin subunits are associated with the formation of intramolecular and intermolecular disulfide bonds, which largely determine gluten structure and network (Shewry et al., 1997). Glutenin protein usually exists in gluten as polymers. According to the solubility in SDS solution, glutenin polymers were divided into SDS-unextractable polymeric protein (UPP) and SDS-extractable polymeric protein (EPP). The percentage of UPP in total glutenin polymer (UPP%) was strongly related to optimal mixing time of dough rheological property and thimble-loaf height (Don et al., 2006). We found that gene silencing of *1Dy12* in Ningmai9 decreased the total HMW-GSs content and led to a decrease in UPP% (Figure 4). It is speculated that this phenomenon results from the inefficient formation of disulfide bonds. The decrease of UPP% reduced the peak time and TIMEX width in dough rheological properties using mixograph, which reflected a decrease in gluten strength.

Solvent retention capacity (SRC) is a solvation examination for wheat flour that is widely applied for evaluating flour functionality in soft wheat breeding in the United States. The functional contributions of different polymeric components in SRC are predicted on the basis of their swelling properties in different diagnostic solvents (Guttieri et al., 2004). LASRC reflected glutenin network and gluten strength, WSRC is associated with all the assessed polymers water-holding capacity (Meera et al., 2011). A low WSRC would be required to make flour for cookies. With the small amount of water needed for a processable dough for cookies, water therefore could be easier to remove during the baking process, leading to a lower-moisture cookie with a prolonged shelf life. Low LASRC value prevents the formation of networks during dough mixing and baking, which resulted in promoting the collapse of cookie structure, yielding cookies with larger diameter and thinner height (Slade et al., 1993). In this study, SRC tests showed that WSRC and LASRC in 1Dy12 deletion mutants were lower than those in the wild type (Figure 5), which improved the snap-cookie quality.

## Data Availability Statement

The original contributions presented in the study are included in the article/Supplementary Material, further inquiries can be directed to the corresponding authors.

## Author Contributions

HM conceived the experiments. PZ developed the mutants. GL performed major experiments. YG supplemented experiments and analyzed the data. HW performed field experiments. HM and YG wrote the manuscript. JC and YW modified the manuscript. All authors have read and agreed on the published version of the manuscript.

## Conflict of Interest

The authors declare that the research was conducted in the absence of any commercial or financial relationships that could be construed as a potential conflict of interest.

## Publisher’s Note

All claims expressed in this article are solely those of the authors and do not necessarily represent those of their affiliated organizations, or those of the publisher, the editors and the reviewers. Any product that may be evaluated in this article, or claim that may be made by its manufacturer, is not guaranteed or endorsed by the publisher.

## Supplementary Material

Supplementary Figure 1 Amplified fragment of *1Dy12*.

Supplementary Table 1 Values of grain hardness, grain diameter (mm), test weight, grain protein content (%), grain gluten content (%), HMW-GS content (mg g^–1^), Glu/Gli, and UPP/(UPP + EPP) in the wild type and mutants (md10 and md10).

Supplementary Table 2 Solvent retention capacity, WSRC, SCSRC, LASRC (%), and SUSRC (%) of flour in the wild type and mutants (md10 and md10).

Supplementary Table 3 Dough rheological properties, peak time (min), peak value (%), peak width (%), right slope (% min^–1^), TIMEX (%) using mixograph, and cookie diameter in wild type and mutants (md10 and md10).

Supplementary Table 4 Yield-related characters, plant height (cm), spike number ha^–1^, kernel number spike^–1^, TKW (g), and yield (kg ha^–1^).

Supplementary File 1 Sequence of DNA and deduced amino acid sequence of *1Dy12* in Ningmai9.
