# Supplementary figures and images for: Premature Termination Codon of 1Dy12 Gene Improves Cookie Quality in Ningmai9 Wheat
Source: Front Plant Sci. 2022 May 12;13:835164. doi: 10.3389/fpls.2022.835164 (PMC9134186; doi:10.3389/fpls.2022.835164)

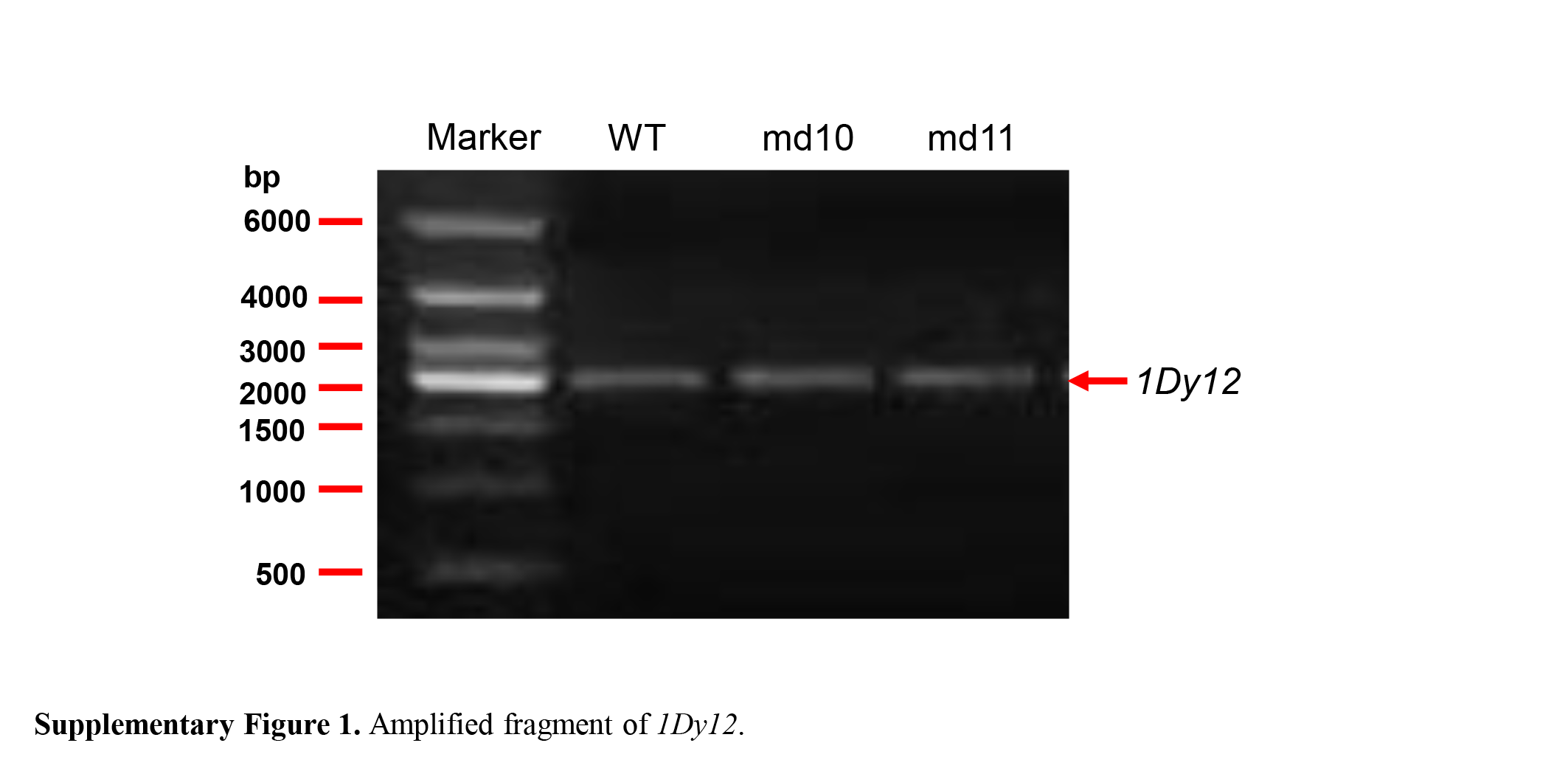

Supplement: Supplementary Figure 1 — Amplified fragment of 1Dy12. [file Image_1.TIF]
